# Non-ST Elevation Myocardial Infarction and Severe Peripheral Artery Disease in a 20-Year-Old with Perinatally Acquired Human Immunodeficiency Virus Infection

**DOI:** 10.1155/2018/7803406

**Published:** 2018-04-01

**Authors:** Purva Sharma, Mohamad Kabach, Samineh Sehatbakhsh, Rashida Tharpe, Shaun Isaac, Robert Chait, Kleper De Almeida

**Affiliations:** JFK Medical Center, University of Miami-Palm Beach Regional GME Consortium, Atlantis, FL, USA

## Abstract

Human immunodeficiency virus (HIV) infection confers an increased risk of cardiovascular disease, including acute coronary syndrome (ACS). Patients with perinatally acquired HIV may be at increased risk due to the viral infection itself and exposure to HAART in utero or as part of treatment. A 20-year-old female with transplacentally acquired HIV infection presented with symptoms of transient aphasia, headache, palpitations, and blurry vision. She was admitted for hypertensive emergency with blood pressure 203/100 mmHg. Within a few hours, she complained of typical chest pain, and ECG showed marked ST depression. Troponin I levels escalated from 0.115 to 10.8. She underwent coronary angiogram showing 95% stenosis of the right coronary artery (RCA) and severe peripheral arterial disease including total occlusion of both common iliacs and 95% infrarenal aortic stenosis with collateral circulation. She underwent successful percutaneous intervention with a drug-eluting stent to the mid-RCA. Patients with HIV are at increased risk for cardiovascular disease. Of these, coronary artery disease is one of the most critical complications of HIV. Perinatally acquired HIV infection can be a high-risk factor for cardiovascular disease. A high degree of suspicion is warranted in such patients, especially if they are noncompliant to their ART.

## 1. Introduction

Human immunodeficiency virus (HIV) infection is associated with an increased risk of cardiovascular disease through various mechanisms including premature atherosclerosis from endothelial inflammation and platelet dysfunction as well as from cART therapy involving protease inhibitors resulting in dyslipidemia and insulin resistance [1]. One of the most important cardiac presentations is coronary artery disease leading to acute coronary syndrome (ACS). Patients with perinatally acquired HIV infection may be at an increased cardiac risk due to a combination of in utero HIV exposure and long-term exposure to cART [2]. We present a case of a 20-year-old African American female who acquired HIV infection through transplacental transmission and presented to us with ACS.

## 2. Case Report

A 20-year-old African American female with HIV infection since birth, acquired by transplacental transmission, presented to the emergency department with symptoms of transient aphasia lasting a few seconds. She described associated headache, palpitations, and blurry vision for the past 24 hours. Apart from HIV infection, she had no other significant past medical history and denied any surgeries in the past. She had no history of alcohol or tobacco use or illicit drug use. On presentation, her blood pressure was 203/100 mmHg and pulse was regular with 118 beats/minute. The rest of her exam was pertinent for tachycardia, palpable thrill, and a displaced point of maximal impulse. Remainder of the physical examination was unremarkable.

**Figure 1:**
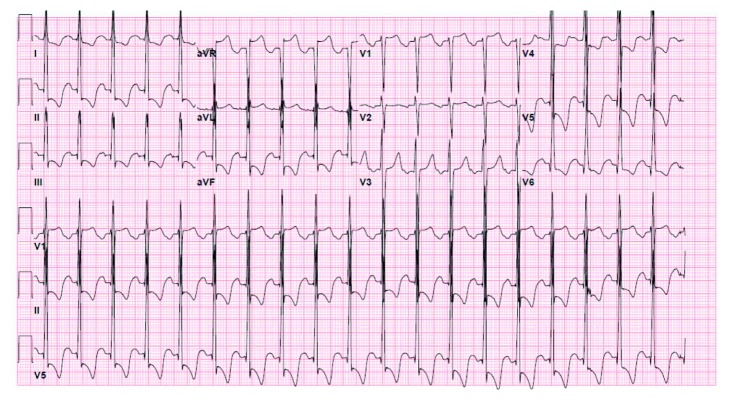
EKG showing ST segment depressions in inferolateral leads.

**Figure 2:**
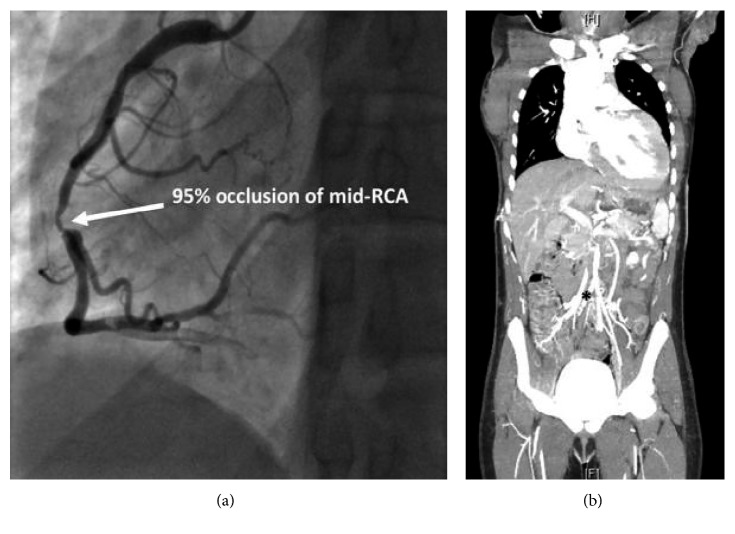
(a) 95% stenosis of the right coronary artery (RCA). (b) 95% infrarenal aortic stenosis with collateral circulation.

Her initial laboratory tests were notable for microcytic anemia (Hb 7.3, MCV 64), hypokalemia (3.4 mEq/L), and elevated creatinine (1.4 mg/dL). She also had an elevated troponin level of 0.115, and her NT-proBNP was 23,245. Her CD4 count was 78 cells/*µ*L, and HIV-1 ribonucleic acid viral load was 9018 copies/ml. Upon presentation, her electrocardiogram (ECG) showed sinus tachycardia with a ventricular rate in the 100 s and some nonspecific ST-T wave changes. Her chest radiograph and CT scan of the brain were normal. Further imaging of the brain such as MRI was not performed. She was admitted to the intensive care unit for hypertensive emergency and was started on an esmolol drip. However, within a few hours of admission, she complained of typical chest pain. A repeat ECG showed marked ST depression in the inferolateral leads, suggestive of ischemia (Figure 1). She was started on ACS protocol with aspirin, clopidogrel, and unfractionated heparin drip. Echocardiogram revealed moderate left ventricular hypertrophy with moderate-to-severe left ventricular dysfunction and ejection fraction (EF) of 30–35%. Her troponin I levels escalated from 0.115 to 10.8. She underwent coronary angiogram which showed 95% stenosis of the right coronary artery (RCA). It also revealed stenosis of the distal aorta. Due to this finding, a peripheral CT angiogram was done for further evaluation of the arterial system which showed severe peripheral arterial disease including total occlusion of both common iliac arteries and 95% infrarenal aortic stenosis with collateral circulation (Figures 2(a) and 2(b)). She underwent successful percutaneous intervention with a drug-eluting stent to the mid-RCA. The patient was discharged on dual antiplatelet therapy, high-intensity statin, a beta blocker and spironolactone, and cART.

## 3. Discussion

Patients with HIV infection are at increased risk for cardiovascular disease [3].

Of these, ACS is now one of the most critical complications of HIV. Since the advent of efficient antiretroviral therapies and the consequent longer patient life span, an increased risk for myocardial infarction has been observed in HIV-infected patients compared with the general population in Western countries [4]. HIV infection leads to chronic immune activation which plays a role in the pathogenesis of HIV-related atherosclerosis and CAD [5]. Post et al. studied the association between HIV infection and subclinical atherosclerosis through CT angiography. They found that coronary artery plaque, especially noncalcified plaque, is more prevalent and extensive in HIV-infected men, independent of CAD risk factors [6]. In our patient, although none of the imaging studies reported atherosclerosis, subclinical atherosclerosis cannot be ruled out. Also, there was a suspicion for fibromuscular dysplasia in our patient, given the findings of the CT angiogram which could be explained by the vascular endothelial dysfunction due to HIV infection. Endothelial dysfunction is known to be one of the important mechanisms by which HIV affects the blood vessels. In a study by Torriani et al., it was shown that the endothelial function improved after starting ART regimens [7].

HIV infection is associated with increased risk of acute myocardial infarction although the mechanism behind this is not clearly understood [8]. Studies have shown that both HIV infection and the concomitant use of ART play a role in increased AMI risk. However, infection with virus itself could be the main cause as studies have shown that viral load suppression results in lower cardiovascular disease risk [9]. HIV-related cardiomyopathy is a significant manifestation of cardiac disease in HIV-infected patients. Left ventricular diastolic dysfunction appears to be the most common manifestation [10], although systolic dysfunction is also observed with moderate frequency, particularly among untreated HIV-infected patients. In a prospective multicenter study of 196 children infected with HIV through vertical transmission, baseline echocardiogram and follow-up every 4 months for 2 years was obtained. The study showed that subclinical cardiac abnormalities such as dilated cardiomyopathy (depressed contractility and dilatation) and inappropriate LV hypertrophy were commonly seen as part of perinatally acquired HIV [11].

In our patient, the systolic function was reduced with ejection fraction of 30%–35%, and Doppler readings were consistent with grade 1 diastolic dysfunction. The etiology of the patient's cardiomyopathy could be attributed to the long-standing hypertension as well as HIV infection.

Our patient had a known history of noncompliance to her HAART medications, and her CD4 count was low at the time of presentation with a high viral load. We could thus hypothesize that the uncontrolled viral replication in our patient was one of the major risk factors for cardiovascular disease. Many studies have shown that adherence to HAART is the critical behavior underlying many of the long-term outcomes for children and adolescents with HIV [12]. Thus, this would be one of the major challenges as seen in our patient and would require counselling the patient and their family.

Another possibility also considered was HIV-related Takayasu's arteritis (TA) which is described by the American College of Rheumatology as a HIV-associated rheumatic disease and can present with ACS. Features that led to this being considered included her age under 40, hypertension, CT imaging that showed diffuse narrowing in the distal aorta, and a markedly elevated ESR/CRP [13]. Our patient did not have aneurysms which, while well described in the literature, are not pathognomonic of TA [14].

## 4. Conclusion

We thus conclude that perinatally acquired HIV infection can be a high-risk factor for CVD. In our patient, the fact that she was noncompliant with her medication also put her at risk for accelerated atherosclerosis. A high degree of suspicion is warranted in such patients, especially if they are noncompliant to their ART. Further studies are needed to assess CVD risk and to establish guidelines on CVD risk management.
